# Axial spondyloarthritis and inflammatory bowel disease: association between disease activity and endothelial dysfunction markers

**DOI:** 10.1007/s00296-021-04940-1

**Published:** 2021-07-08

**Authors:** Hanna Przepiera-Będzak, Katarzyna Fischer, Marek Brzosko

**Affiliations:** 1 Department of Rheumatology, Internal Medicine, Geriatrics and Clinical Immunology, Pomeranian Medical University in Szczecin, Unii Lubelskiej 1, 71-252 Szczecin, Poland; 2 Independent Laboratory of Rheumatic Diagnostics, Pomeranian Medical University in Szczecin, Szczecin, Poland

**Keywords:** axSpA, IBD, IL-6, IL-18, ADMA, ET-1

## Abstract

**Objective:**

We aimed to assess patients with axial spondyloarthritis (axSpA) and inflammatory bowel disease (IBD) for disease activity and serum markers of endothelial dysfunction**.**

**Methods:**

We studied 161 patients (123 males, 38 females) with axSpA: 153 with ankylosing spondylitis and 8 with non-radiographic axSpA, and 30 healthy controls (HC). We collected: age; sex; disease duration; extra-articular symptoms (IBD and acute anterior uveitis), comorbidities; human leukocyte antigen B27 status; and treatment. We measured serum interleukin (IL)-6, interleukin-18, IL-23, vascular endothelial growth factor (VEGF) epidermal growth factor (EGF), asymmetric dimethylarginine (ADMA), endothelin-1 (ET-1), and fetuin-A levels.

**Results:**

IBD was diagnosed in 19 (11.8%) patients with axSpA. Compared to patients with axSpA without IBD, those with IBD had higher serum C-reactive protein (CRP) level (*p* = 0.05), erythrocyte sedimentation rate (ESR) (*p* = 0.005), and serum ET-1 levels (*p* = 0.01). In patients with axSpA and IBD, ET-1 levels correlated positively with CRP level (*p* = 0.006) and ESR (*p* = 0.02), and ADMA levels with visual analog scale scores (*p* = 0.01). Patients with axSpA and IBD had higher serum levels of IL-6 (*p* = 0.01), IL-18 (*p* = 0.005), and ADMA (*p* = 0.01) and lower serum levels of fetuin-A (*p* = 0.01) than did controls.

**Conclusions:**

Patients with axSpA and IBD had higher levels of disease activity, as assessed by ESR and CRP and ET-1 levels, than did patients with axSpA without IBD. Compared to HC, patients with axSpA and IBD had increased IL-18, ADMA levels and decreased fetuin-A level.

## Introduction

Axial spondyloarthritis (axSpA) is a seronegative spondyloarthritis (SpA) associated with the presence of human leukocyte antigen B27 (HLA-B27). The definition for axSpA covers both non-radiographic disease and radiographic disease, which is also known as ankylosing spondylitis (AS) [1]. In addition, axSpA exhibits different extra-articular symptoms, such as inflammatory bowel disease (IBD) and acute anterior uveitis (AAU) [2–4].

Certain proinflammatory cytokines [interleukin (IL)-6 and IL-23], cytokines involved in angiogenesis, and cytokines and adhesion molecules that activate and deregulate endothelial function [IL-18, vascular endothelial growth factor (VEGF), epidermal growth factor (EGF), asymmetric dimethylarginine (ADMA), endothelin-1 (ET-1) and fetuin-A] are involved in the inflammatory process of AS [5–11]. Vascular abnormalities and endothelial dysfunction play a role in IBD [4, 7, 12–14]. In axSpA patients, decreased serum fetuin-A could result in a decrease in its protective role in ectopic calcification, and in that way could influence on endothelial dysfunction [7]. ET-1, a proinflammatory peptide produced mainly by endothelial cells, contributes to inflammation, fibrosis and vascular hypertrophy. In axSpA patients abnormal serum levels of ET-1 were observed, but its role in IBD was not clearly defined [7, 8]. ADMA is an endogenous inhibitor of nitric oxide, and increased levels of this molecule are associated with reduced nitric oxide synthesis and vascular dysfunction. Increased ADMA levels obtained in AS patients without CV risk factors and in IBD patients suggests that nitric oxide metabolism is impaired in AS and IBD [8–10, 13, 14].

Moreover, axSpA is genetically and pathophysiologically linked to IBD [3, 4, 7].

Antineutrophil cytoplasmic antibodies (ANCA) that are positive in a significant proportion of patients with IBD, more prevalent in those with ulcerative colitis (UC) than with Crohn’s disease (CD), might be helpful in subtyping UC from CD [15].

This study aimed to assess patients with axSpA and IBD for disease activity, certain serum proinflammatory cytokines, and cytokines and adhesion molecules involved in endothelial dysfunction.

## Materials and methods

The ethics committee of the Pomeranian Medical University in Szczecin approved this study (KB-0012/106/10; 27SEP2010), and all patients provided informed consent. We studied 161 Caucasian patients with axSpA and recorded data on age; sex; disease duration; diagnosis of extra-articular symptoms: IBD (CD or UC) and AAU; comorbidities; C-reactive protein (CRP) level; erythrocyte sedimentation rate (ESR); positivity for HLA-B27 and ANCA; and treatment. Patients were diagnosed with axSpA according to the Assessment of Spondyloarthritis International Society (ASAS) classification criteria for axSpA [16]. For the diagnosis of AS, the modified New York criteria were used [17].

The diagnosis of IBD was made by a gastroenterologist based on clinical presentation, colonoscopy and histopathological examination. IBD activity was assessed by a gastroenterologist using Crohn’s disease activity index (CDAI) in patients with CD and Mayo score disease activity index in patients with CU.

The patient’s pain due to the disease at the time of examination was assessed using a visual analog scale (VAS). We assessed the disease with the Bath Ankylosing Spondylitis Disease Activity Index (BASDAI) [18]. The Ankylosing Spondylitis Disease Activity Score (ASDAS) was assessed based on ESR and calculated using an online calculator available at the ASAS website.

We studied serum levels of IL-6, IL-23, IL-18, VEGF, EGF, ADMA, ET-1, and fetuin-A of the first 81 patients with axSpA. The control group consisted of 30 healthy volunteers. For the estimation of ADMA level, serum was stored at − 80 °C until analysis using a sensitive sandwich ELISA method called Human ADMA Immunoassay ELISA kit (Wuhan EIAab Science Co., Ltd.). Meanwhile, ANCA positivity was detected by indirect immunofluorescence. Sera were scored as p-ANCA/c-ANCA or negative. Serum was stored at − 80 °C until analysis for IL-6, IL-23, IL-18, VEGF, EGF, ET-1, and fetuin-A using a sensitive sandwich ELISA method using the kits presented in our previous paper [7].

Data distributions were evaluated using a Kolmogorov–Smirnov test. Data are presented as means (SD) and medians (Q1, Q3). The R values of correlations were also determined. The groups were compared using a Student’s t test, Mann–Whitney U test, and Kruskal–Wallis test. The parameters were evaluated using a Pearson’s chi-squared test (*χ*^2^), logistic regression analysis, and stepwise analysis, and a *p* < 0.05 was considered statistically significant. All statistical data were analyzed using STATISTICA 8.0 (StatSoft, Inc., Tulsa, USA).

## Results

The baseline characteristics of all the patients are shown in Table 1. We studied 161 patients (123 males, 38 females) with axSpA. A total of 153 patients (119 males, 34 females) had AS, whereas 8 (4 males, 4 females) had non-radiographic axSpA. Of the 161 patients with axSpA, 19 (11.8%) had IBD (18—ulcerative colitis, 1—Crohn's disease), and 15 (78.9%) of them were males (Table 1).

**Table 1:** Clinical characteristics of axial spondyloarthritis patients with and without inflammatory bowel disease, and healthy controls

Assessed parameter	Patients with axSpA and IBD (*n* = 19) Mean ± SD Median (Q1, Q3)	Patients with axSpA without IBD (*n* = 142) Mean ± SD Median (Q1, Q3)	Healthy Controls (*n* = 30)
Age, years	44.6 ± 10.6	46.4 ± 13.3	43.5 ± 9.4
axSpA disease duration, years	10.4 ± 9.5	12.1 ± 9,4	0
IBD disease duration, years	8.47 ± 6.9	0	0
IBD diagnosis preceded axSpA diagnosis, *n* (%)	6 (31.6)	0	0
axSpA diagnosis preceded IBD diagnosis, *n* (%)	10 (52.6)	0	0
Nonradiographic axSpA, *n* (%)	2 (10.5)	6 (4.2)	0
Ankylosing spondylitis, *n* (%)	17 (89.5)	136 (95.8)	0
HLA-B27, *n* positive/n assessed (%)	18/19 (94.7)	109/115 (94.7)	0
ANCA, *n* positive/n assessed (%)	1/5 (20)	10/62 (16.1)	0/30 (0)
AAU, *n* (%)	7 (36.8)	35 (24.6)	0
Hypertension, *n* (%)	5 (26.3)	33 (23.2)	0
Diabetes, *n* (%)	0 (0)	4 (2.8)	0
IHD, *n* (%)	3 (15.7)	10 (7.0)	0
Smoking, *n* (%)	2 (10.5)	14 (9.8)	0
NSAIDs only, *n* (%)	0 (0)	36 (25.3)	0
Sulfasalazine, *n* (%)	12 (63.1)	76 (53.2)	0
Adalimumab, *n* (%)	6 (31.6)	6 (4.2)	0
Golimumab, *n* (%)	0 (0)	1 (0.7)	0
Infliximab, *n* (%)	2 (10.5)	6 (4.2)	0
Methotrexate, *n* (%)	2 (10.5)	17 (11.9)	0
Prednisone, n (%)	2 (10.5)	0 (0)	0

*AAU* acute anterior uveitis, *axSpA* axial spondyloarthritis, *HLA-B27* human leukocyte antigen B27, *IBD* inflammatory bowel disease, *IHD* ischemic heart disease, *NSAIDs* nonsteroidal anti-inflammatory drugs, *n* number

Patients with axSpA and IBD had higher serum levels of IL-6 (*p* = 0.01), IL-18 (*p* = 0.005), and ADMA (*p* = 0.01) and lower serum levels of fetuin-A (*p* = 0.01) than did the healthy controls. Serum ET-1 levels (*p* = 0.02) were also higher in patients with axSpA and IBD than in those without IBD (Table 2). Additionally, serum ET-1 levels correlated positively with ESR (*R* = 0.82; *p* = 0.02) and CRP level (*R* = 0.89; *p* = 0.006) (data not shown).

**Table 2:** Comparison of markers of disease activity and serum levels of selected cytokines between axial spondyloarthritis patients with and without inflammatory bowel disease (p^1^) and between axial spondyloarthritis patients with inflammatory bowel disease and healthy controls (p^2^)

Assessed Parameter	Patients with axSpA and IBD Mean ± SD Median (Q1, Q3)	Patients with axSpA without IBD Mean ± SD Median (Q1, Q3)	P^1^	Healthy Controls Mean ± SD Median (Q1, Q3)	P^2^
VAS pain, mm	64.2 ± 26.3	55.1 ± 26.6	0.08*	0.0	–
BASDAI	5.7 ± 2.9	5.3 ± 2.5	0.2*	0.0	-
ASDAS-ESR	3.1 (2.2, 4.1)	2.4 (1.8, 3.2)	0.06**	0.0	–
CRP (mg/l)	17.2 (2.1, 80.0)	7.3 (2.8, 16.2)	0.05**	0.0	–
ESR (mm/h)	28.0 (11.0, 58.0)	14.0 (6.0, 32.0)	0.005**	9.0 (2.0, 16.0)	0.0008**
IL-6 (pg/ml)	2.7 (1.5, 5.6)	4.1 (1.8, 7.9)	0.2**	1.15 (0.6, 1.5)	0.01**
IL-18 (pg/ml)	357.3 (166.7, 402.7)	251.8 (207.7, 375.1)	0.4**	198.9 (165.1, 271.5)	0.005**
IL-23(pg/ml)	0.0 (0.0, 0.3)	0.3 (0.0, 2.8)	0.2**	0.0 (0.0, 0.0)	0.2**
VEGF (pg/ml)	386.5 (332.7, 570.0)	396.1 (220.0, 680.0)	0.2**	270.0 (180.0, 445.0)	0.2**
EGF (pg/ml)	66.0 (40.0, 122.0)	128.0 (86.0, 220.0)	0.2**	81. 0 (38.0, 134.0)	0.2**
ADMA (nmol/ml)	13.39 ± 2.2	14.7 ± 4.5	0.2*	9.53 ± 4.08	0.01*
ET-1 (pg/ml)	1.40 ± 0.51	1.10 ± 0.35	0.02*	1.47 ± 0.57	0.3*
Fetuin-A (µg/ml)	527.2 ± 195.8	608.7 ± 143.1	0.08*	709.1 ± 169.3	0.01*

*ADMA* asymmetric dimethylarginine, *ASDAS-ESR* Ankylosing Spondylitis Disease Activity Score, *axSpA* axial spondyloarthritis, *BASDAI* Bath Ankylosing Spondylitis Disease Activity Index, *EGF, CRP* C-reactive protein; epidermal growth factor, *ESR* erythrocyte sedimentation rate, *ET-1* endothelin-1, *IBD* inflammatory bowel disease, *IL-6* interleukin-6, *IL-18* interleukin-18, *IL-23* interleukin-23, *VAS pain* visual analogue scale of patient’s pain, *VEGF* vascular endothelial growth factor

Statistical analysis: *Student's *t* test, ** Mann–Whitney *U* test

The majority of patients had moderate IBD activity estimated by a gastroenterologist using clinical indexes. Moreover, patients with axSpA and IBD demonstrated a higher disease activity, as assessed by CRP level (*p* = 0.05) and ESR (*p* = 0.005), than did those without IBD. No differences in terms of VAS, BASDAI, and ASDAS-ESR scores (all *p* > 0.05) were observed (Table 2). Increased serum IL-18 (*p* = 0.05), ADMA (*p* = 0.02), and CRP levels (*p* = 0.02) and ESR (*p* = 0.01) and decreased serum fetuin-A level (*p* = 0.02) were associated with an increased risk of axSpA and IBD (Table 3).

**Table 3:** A logistic regression model of the OR of selected cytokines serum levels and markers of disease activity as predictors of axial spondyloarthritis and inflammatory bowel disease

Covariates	Patients with axSpA and IBD	
	OR (95% CI)	*p*
IL-6 ≥ 6.64 pg/ml	2.25 (0.17–29.05)	0.5
IL-18 ≥ 227.45 pg/ml	1.25 (0.23–6.92)	0.7
ADMA ≥ 9.885 nmol/ml	30.3 (1.50–609.8)	0.02
ET-1 ≤ 1.081 pg/ml	1.08 (0.10–11.52)	0.94
Fetuin-A ≤ 608.5 µg/ml	9.58 (1.47–62.17)	0.01
CRP ≥ 10 mg/L	3.13 (1.12–8.73)	0.02
ESR ≥ 60 mm/h	5.14 (1.35–19.62)	0.01

*ADMA* asymmetric dimethylarginine, *ET-1* endothelin-1, *IBD* inflammatory bowel disease, *IL-6* interleukin-6, *IL-18* interleukin-18, *OR* odds ratio, *p* p value

All the ANCA tests were scored as p-ANCA (Table 1). Positivity for ANCA was not associated with an increased risk of axSpA and IBD (odds ratio, 1.24; 95% confidence interval, 0.13–11.74; *p* = 0.85).

Furthermore, in patients with axSpA and IBD, serum IL-6 levels negatively correlated with serum fetuin-A levels (*R* = − 0.82; *p* = 0.04), while serum ADMA levels positively correlated with VAS scores (*R* = 0.89; *p* = 0.01) (data not shown).

Disease duration, cigarette smoking, and comorbidities did not increase the risk of IBD in patients with axSpA (all *p* > 0.05). Regarding cigarette smoking and comorbidities, serum levels of selected cytokines showed no differences in patients with axSpA (all *p* > 0.05).

## Discussion

We assessed patients with axSpA and IBD for disease activity and serum markers of endothelial dysfunction.

The incidence of IBD among patients with axSpA in our study was similar to those presented in other studies [2–4, 7]. In our study, patients with axSpA and IBD demonstrated a higher level of disease activity, as assessed by the ESR, CRP, and IL-6 levels, than did those without IBD. Additionally, both increased CRP level and ESR were associated with increased risk of axSpA and IBD, which was consistent with the observations of other authors [2–4]. Comparison of axSpA patients with and without IBD showed no difference in clinical disease activity as assessed by VAS, BASDAI, and ASDAS-ESR scores, suggesting that the differences in CRP and ESR values in these patient groups were due to IBD activity rather than arthritis activity.

IL-18 is a cytokine associated with endothelial dysfunction. In our previous study, patients with SpA who had high serum IL-18 levels were associated with an increased risk of IBD compared to that of controls [7]. In the current study, higher serum IL-18 levels were observed in patients with axSpA and IBD than in controls and were associated with increased risk of axSpA and IBD. Therefore, deregulation of endothelial function by IL-18 could be involved in the inflammatory process of axSpA and IBD.

Another marker of endothelial dysfunction, ADMA, is an endogenous inhibitor of nitric oxide. Increased serum ADMA levels in patients with AS correlates with disease activity, suggesting that the metabolism of nitric oxide is impaired in AS [8–10]. Other researchers observed higher serum ADMA levels in the IBD group than in the control group [13, 14]. In our study, patients with axSpA and IBD had higher serum levels of ADMA than did controls, and ADMA correlated positively with the VAS. In addition, increased serum ADMA levels were associated with an increased risk of axSpA and IBD. Therefore, the findings of this study suggest that patients with axSpA and IBD show enhanced ADMA production, which might be associated with oxidative stress and vascular dysfunction.

Meanwhile, ET-1 is a proinflammatory peptide produced mainly by endothelial cells. Sari et al. [8] found that plasma ET-1 levels were similar in patients with AS and in controls. In our previous study, serum ET-1 levels were lower in patients with AS than in controls [7]. In the current study, serum ET-1 levels were higher in patients with axSpA and IBD than in those without IBD, and ET-1 levels correlated positively with disease activity assessed by the ESR and CRP levels. However, the influence of ET-1 level on endothelial dysfunction in patients with axSpA and IBD requires further investigation.

Fetuin-A is important in physiological and pathological mineralization. In our previous study, patients with SpA and those with AS had lower serum fetuin-A levels than those of controls [7]. In the current study, patients with axSpA and IBD were confirmed to have lower serum fetuin-A levels compared to controls, and serum fetuin-A level was negatively correlated with IL-6 level. Additionally, decreased serum fetuin-A levels increased the risk of axSpA and IBD. Therefore, decreased serum fetuin-A levels in patients with axSpA and IBD could decrease protection against ectopic calcification and increase the risk of endothelial dysfunction.

As patients with axSpA and IBD had no differences in VAS score, BASDAI score, and ASDAS-ESR when compared to those with axSpA without IBD, we can conclude that the higher CRP level, ESR, and serum ET-1 level in patients with axSpA and IBD were due to IBD activity. Moreover, in patients with axSpA and IBD, levels of serum markers of endothelial dysfunction differed from those in controls; specifically, compared to controls, patients with axSpA and IBD had increased serum IL-18 and ADMA levels and decreased serum fetuin-A level.

## Conclusions

Comparison of axSpA patients with and without IBD showed no difference in arthritis disease activity as assessed by VAS, BASDAI, and ASDAS-ESR scores. Patients with axSpA and IBD had higher levels of disease activity, as assessed by ESR and CRP and ET-1 levels, than did patients with axSpA without IBD, suggesting that the differences were due to IBD activity rather than arthritis activity. Compared to healthy controls, patients with axSpA and IBD had signs of endothelial dysfunction assessed by increased IL-18, ADMA levels and decreased fetuin-A level.

## Data Availability

All data available in the article.
